# Oral cancer in Hungary: An epidemiological profile (2015–2019)

**DOI:** 10.1371/journal.pone.0327566

**Published:** 2025-07-03

**Authors:** Amr Sayed Ghanem, Laszlo Trefan, Ildikó Márton, Petra Fadgyas-Freyler, Attila Csaba Nagy, Marianna Móré

**Affiliations:** 1 Department of Health Informatics, Faculty of Health Sciences, University of Debrecen, Debrecen, Hungary; 2 Department of Biochemistry and Molecular Biology, Faculty of Medicine, University of Debrecen, Debrecen, Hungary; 3 National Health Insurance Fund, Budapest, Hungary; 4 Department of Health Policy, Corvinus University of Budapest, Budapest, Hungary; 5 Faculty of Health Sciences, Institute of Social and Sociological Sciences, University of Debrecen, Nyíregyháza, Hungary; University of the Republic Uruguay: Universidad de la Republica Uruguay, URUGUAY

## Abstract

**Objective:**

Hungary is among countries with the highest standardised rate oral and mouth cancers.

This first national analysis aimed to quantify age-standardized oral cancer incidence in Hungary from 2015 to 2019 by sex and age, determine the median interval from diagnosis to death, and evaluate comorbidity prevalence and its prognostic impact.

**Methods:**

We used real-world data from the Hungarian National Health Insurance Fund database. Adjusted, standardised incidence- and hospital readmission rates were calculated; time between diagnosis and death and effect of comorbidities coexisting with oral cancer were analysed.

**Results:**

Generally, incidence and hospitalisation rates decreased in the studied period, for males and older age group both rates were higher. From year 2017 for incidence rates significant differences were found between a particular region and the other regions; and hospitalisation rates differed significantly for three regions from other regions. Almost half (46.3%) of the patients died within a year after diagnosis, mortality rates were 31.8% for males and 14.5% for females. Existence of oral cancer indicated 5.20 (95% confidence interval, 5.03,5.38), 5.84 (95% confidence interval, 5.64,6.04) odd ratios for mortality by two models. Number and relative risk of comorbidities were higher among patients diagnosed with oral cancer than in the control population.

**Conclusion:**

The study highlighted progress in reducing oral cancer incidence rates in Hungary, however significant challenges remain in reducing mortality rates and improving survival within the first year of diagnosis. The study also showed oral cancer a serious burden in the country, especially for males.

## Introduction

Oral squamous cell carcinoma – a prevalent malignancy in the head and neck region – represents a significant health burden [1]. Globally, the incidence of oral cancer (OC) has seen a significant increase from 1990 to 2019, rising from approximately 175,630 cases to 373,100 cases, with the Age-Standardized Incidence Rate (ASIR) slightly increasing from 4.28 to 4.52 per 100,000 individuals [2]. This rise has been observed across both sexes, with male cases rising from 120,660–243,190, and female cases from 54,970–129,910, indicating a persistent gender disparity in incidence rates. Notably, the increase in incidence rates is influenced by changes in Socio-Demographic Index (SDI) levels, with the most pronounced increase in the low-middle SDI regions, where ASIR rose from 5.88 to 6.65 [2]. Trends in Europe exhibit a similar pattern. The number of new cases in this continent rose from approximately 59,568 in 1990–86,920 in 2019, representing a 45% increase [3].

Romania and Hungary are among European countries with the highest ASIR for OCs [4]. For context, the EU-27 average ASIR for lip and oral cavity cancer in 2019 was 5.3 per 100 000, whereas Hungary’s ASIR reached 7.4 per 100 000 in men and 2.9 per 100 000 in women, equating to a combined rate of about 5.0 per 100 000, placing it above the EU average [3]. Interestingly, while the EU-27 average ASIR for lip and oral cavity cancer declined from 6.2 to 5.3 per 100 000 between 1990 and 2019, Hungary’s combined ASIR remained essentially unchanged at around 5.0 per 100 000, continuing to exceed the regional benchmark and ranking among the highest in Eastern Europe [3].

Historical data highlighted a significant 250% increase in cases of OC in Hungary from 1975 to 1999, followed by a period of slight fluctuation but overall stability from the dawn of the millennium to 2006, with cases decreasing from 3,894 in 2001–3,686 in 2006 [5–7].

OC and OPC are predominantly diagnosed in individuals over 40 with the average age at diagnosis around 60, however it is increasingly found in younger patients. Historically, OC affected men at a rate six times higher than women, but recent trends have narrowed this ratio to 2:1 worldwide and also in Hungary [8].

Despite the changing demographics, OC can be characterised by a five-year survival rate of around 50%, attributed to its aggressive nature and tendency for late-stage discovery, often after metastasizing into neck lymph nodes.Unfortunately, younger patients, despite early diagnosis and fewer traditional risk factors, exhibit a particularly unfavourable clinical outcome [9].

Using data from the Hungarian National Health Insurance Fund (NHIF), this study represents the first comprehensive national analysis of oral cancer in Hungary, aiming to quantify the age-standardized incidence from 2015 to 2019 stratified by sex and age group, determine the median interval from diagnosis to death, and evaluate the prevalence and prognostic significance of key comorbidities, included here in recognition of the growing evidence that comorbidity burden profoundly shapes cancer treatment tolerance, survival outcomes, and the need for integrated patient care.

## Materials and methods

### Data source and study design

This study utilised electronic patient records (claim data) from the NHIF between 2015 and 2019. Due to the fact that Hungary has one single payer for health services, and almost the whole population is covered, the study gives a comprehensive overview about the OC situation in the country. Study population was defined with ICD-10 codes [10]. (Supplementary) S1 Table shows the cancer types related to these codes, so that every person with at least one occurrence of any form of OC in the providers’ report during the study period belonged to the case group. For each person affected by OC, five persons (with non-OC diagnosis) were selected and matched based on exact age, gender and county level place of residence, resulting in a matched case control study.

Incidence was defined as first occurrence of a given type of OC diagnosis with an observation time of one year prior without the diagnosis of OC.

NHIF stores data related to hospital services where admission- and discharge dates are available. Hospitalisation was defined as when a patient had at least one OC related hospital admission and when both admission- and discharge dates were within our study period. Readmission was defined based on the hospitalised patients’ unique insurance number where for each OC related admission, admission- and discharge dates fell into our study period.

Mortality data were available from the National Registry of Residence and incorporated into NHIF data.

To extend the study’s scope beyond demographic factors, comorbidity variables were incorporated as potential contributors to OC development and outcome. These included conditions related to alcohol consumption, as well as to diseases of the digestive-, cardiovascular- and respiratory systems, and stomatological conditions. Tobacco use was not available in the data used. Comorbidities were defined as at least one occurrence of a given ICD 10 code as listed in the (supplementary) S2 Table. The cumulative impact of multiple comorbidities was also noted and considered as a contributor to a possibly worse outcome of OC.

### Data management

Data were accessed for research purposes between 1^st^ of March 2023 and 1^st^ of November 2023 of NHIF. In concordance with stringent data privacy and information governance protocols, this study ensured the complete anonymity of patient data, analyzing anonymized records on the servers of the NHIF. Patient level demographic data as well as data for inpatient care and outpatient specialist care were handled in Oracle relational databases [11], queries were written in structured query language (SQL) [11]. Approval was granted by the Ethics Committee of the University of Debrecen (5609–2020).

### Ethics approval and consent to participate

The studies involving humans were approved by Ethics of Committee of the University of Debrecen (6921−2024). The studies were conducted in accordance with the local legislation and institutional requirements. Written informed consent for participation was not required from the participants or the participants’ legal guardians/next of kin in accordance with the national legislation and institutional requirements.

### Statistical analysis

In this study, standardised rates for incidence and hospitalisation were computed, incorporating variables such as gender, age groups, and geographical location of the habitat of patients at the county level. Direct standardization was employed [12], utilising the Hungarian population’s average demographics by gender, age, and geographical location at county level for each year, as provided by the Hungarian Central Statistical Office. Adjusted standardised rates for incidence and hospitalisation were then derived for each stratum, including distinctions by year, gender, age groups (under 65, and 65 or older), and geographical divisions (19 counties, the capital city, as well as seven NUTS 2 regions) [13] based on the patient’s postal code of home address; e.g., adjusted rates for geographical level, which was finally the Euro regions, were adjusted according to gender and age groups. Statistical analyses employed logistic regression for death odds ratios [12], the χ2 test for associations between two variables [12], and the Shapiro or Kolmogorov test for data normality, with the Kolmogorov-Smirnov test [12] for non-normally distributed data comparisons. All analyses were conducted using R statistical software (version 3.6.1) [14].

## Results

### Study population

From 2015 to 2019, Hungary, we found in the administrative database of NIHF 22,702 persons recorded with a diagnosis of OC, 113,510 control person were matched to these cases without OC diagnosis added up to a study population 136,212. The annual number of cases peaking at 6,781 patients in 2015 and reducing to a minimum of 3,735 patients in 2018 (Table 1). Patient numbers varied in other years: 4,248 in 2016, 3,938 in 2017, and slightly increasing to 4,000 in 2019 (Table 1). The gender distribution showed a predominance of male patients (13,337) over females (9,365).

**Table 1:** Number of annual oral cancer cases in Hungary from 2015 to 2019.

Year	Number of oral cancer cases
2015	6,781
2016	4,248
2017	3,938
2018	3,735
2019	4,000

**Fig 1:**
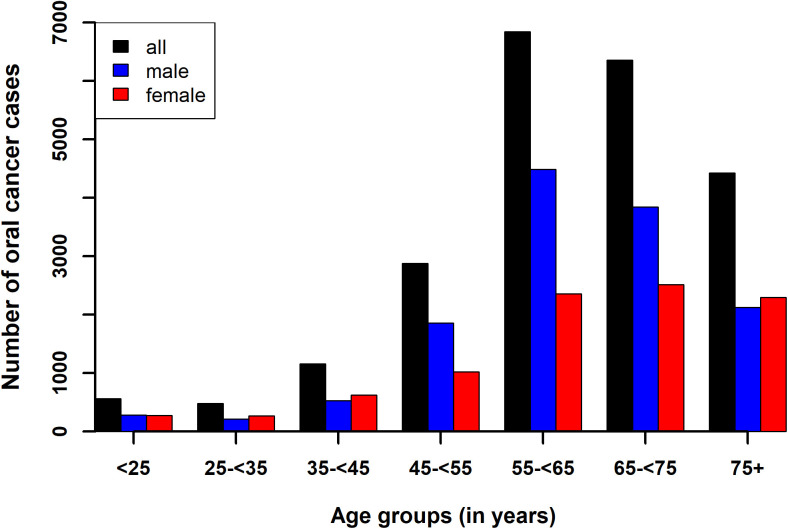
Age distribution of patients with oral cancer in Hungary from 2015 to 2019 for males, females and all patients.

**Fig 2:**
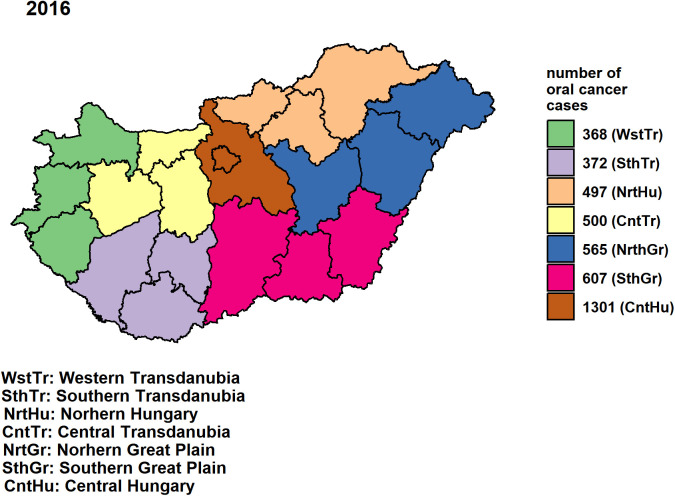
Number of oral cancer cases in Hungary in 2016 in the different NUTS 2 regions (the same colours depict the same regions).

Fig 1 shows the age distribution of patients with OC in different age groups and genders (further details can be found in the supplementary S3 Table). The age range of patients stretched from less than 1 year to 104 years, with a mean age of 56 (SD: 11.1, median: 62.6) years (Table 3). Specifically, the female mean- and median ages were slightly higher than those of males, with significant statistical differences (p < 0.001) in age distribution between genders (Table 2) and a notable association (p < 0.001) between age groups of <25-, 25- < 35-, 35- < 45-, 45- < 55-, 55- < 65-, 65- < 75-,75 + years and gender (S3 Table). Fig 2 shows a typical geographical distribution of OC cases in Hungary in year 2016.

**Table 2:** Summary statistics (in years) of age of all-,male- and female patients with oral cancer cases in Hungary from 2015 to 2019.

	Minimum	Maximum	Mean	Standard deviation	Median
**All cases**	0.0	104.0	64.0	11.1	62.6
**Male**	0.0	102.0	62.3	13.6	63.0
**Female**	0.0	104.0	63.1	16.4	65.0

**Table 3:** Summary statistics (in years) of age of all-,male- and female patients hospitalised with oral cancer in Hungary from 2015 to 2019.

	Minimum	Maximum	Mean	Standard deviation	Median
**All cases**	3.2	97.2	63.5	11.1	63.0
**Male**	8.1	93.2	62.5	10.3	62.2
**Female**	3.2	97.2	65.7	12.4	65.3

### Incidence results

**Fig 3:**
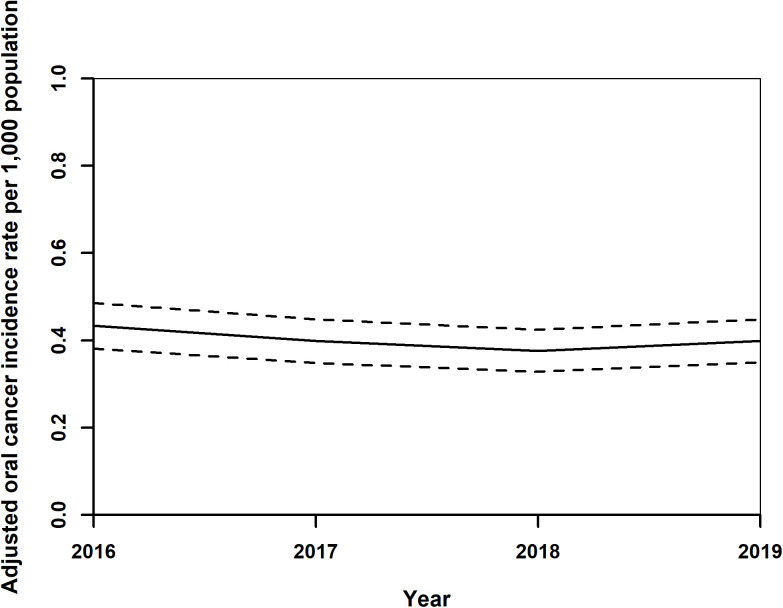
Adjusted standardised oral cancer incidence rates in Hungary from 2016 to 2019.

**Fig 4:**
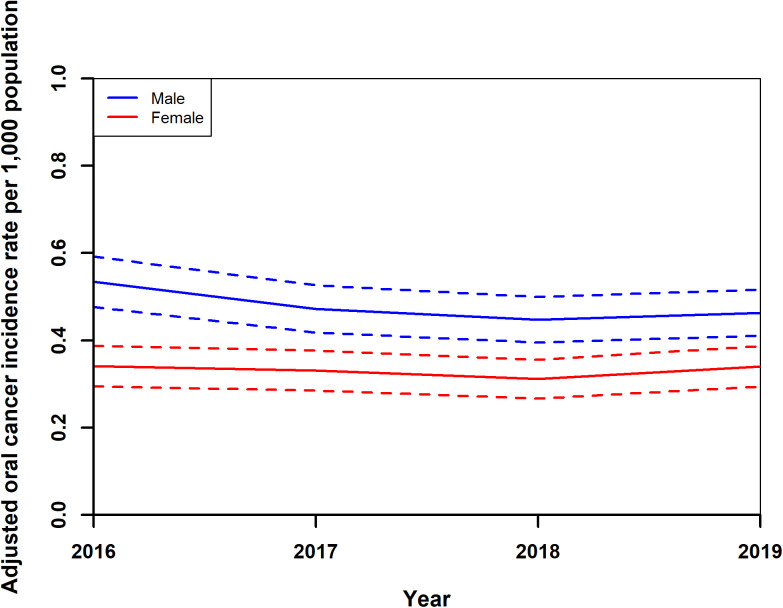
Adjusted standardised oral cancer incidence rates of males and females in Hungary from 2016 to 2019.

**Fig 5:**
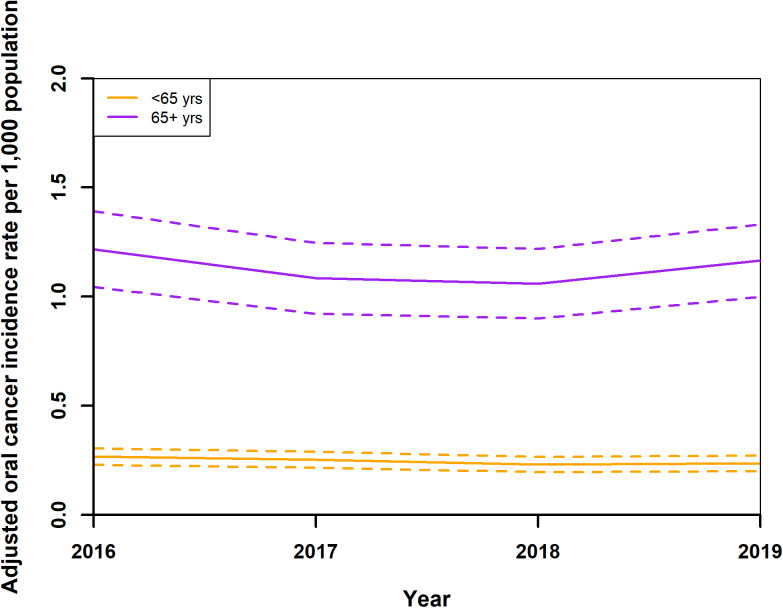
Adjusted standardised oral cancer incidence rates of age group younger than 65 (<65 yrs)- and older than 65 years (5+ yrs) in Hungary from 2016 to 2019.

**Fig 6:**
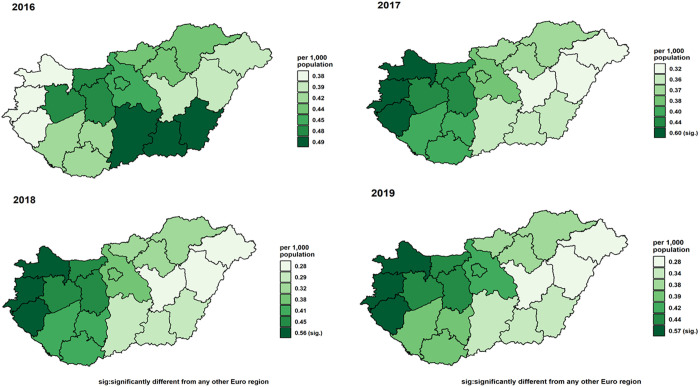
Adjusted standardised oral cancer incidence rates in the seven NUTS 2 regions of Hungary in 2016, 2017, 2018, and 2019 (the same colours depict the same regions).

Incidence data were calculated from year 2016. Adjusted standardised OC incidence rates showed a notable trend from 2016 to 2019. Initially, rates decreased from 0.43 (0.38,0.49) in 2016 to 0.37 (0.33,0.42) per 1,000 population in 2018, before slightly increasing to 0.40 (0.35,0.45) in 2019 (Fig 3). During this period, adjusted standardised rates for both genders also exhibited a decline, from 0.53 (0.48,0.59) for males and 0.34 (0.29,0.39) for females in 2016, to 0.45 (0.39,0.50) for males and 0.31 (0.27,0.35) for females in 2018, with a minor uptick in 2019 to 0.46 (0.41,0.51) for males and 0.34 (0.29,0.39) for females (Fig 4). The analysis by age groups demonstrated a decrease in adjusted standardised rates from 1.22 (1.04,1.39) for those 65 and older and 0.27 (0.231,0.31) for individuals under 65 in 2016, to 1.06 (0.90,1.22) and 0.23 (0.20,0.27), respectively, in 2018, with a slight increase in 2019 to 1.16 (1.00,1.33) and 0.24 (0.20,0.27) (Fig 5). Geographical analysis across Hungarian NUTS2 regions revealed a general decrease in rates from 2016 to 2019 except in Western Transdanubia and certain regions where rates slightly increased by the study’s end (Fig 6). Standardised rates of Western Transdanubia were significantly different to any other NUTS 2 regions from year of 2017–2019 (Fig 6).

### Analysis of hospitalised patients

**Fig 7:**
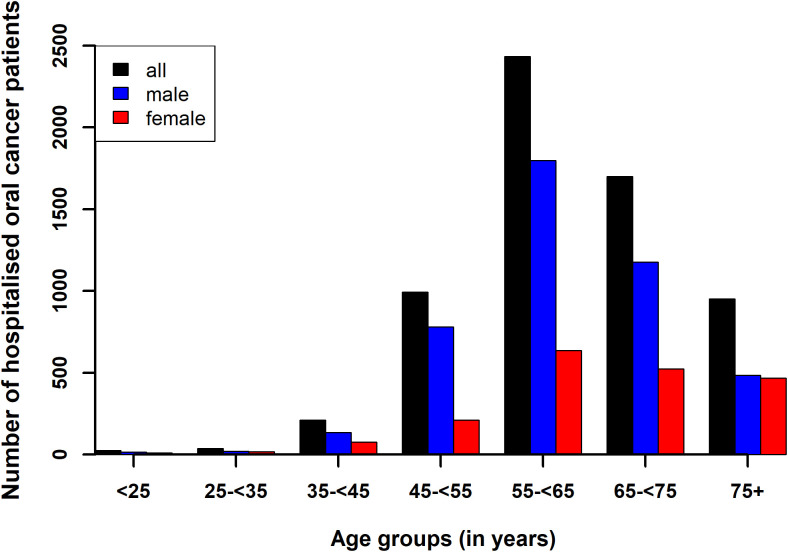
Age distribution of patients hospitalised with oral cancer in Hungary from 2015 to 2019 for male-, female- and all patients.

Out of the 22,702 persons with OC diagnosis, during the study period, 6,356 patients were hospitalised due to OC, of which were 4,412 (69.4%) males and 1,944 (30.6%) females. Fig 7 showed the age distribution of patients hospitalised with OC in different age groups and genders (further details can be found in the S4 Table). Patient ages ranged from 3.19 to 97.3 years, with a mean of 63.5 (SD: 11.1, median: 63.0) years. Male patients showed a narrower age range of 8.08 to 93.2 years, mean age of 62.5 (SD: 10.3, median: 62.2) years. Female patients exhibited ages from 3.2 to 97.2 years, a mean of 65.7 (SD: 2.4, median: 62.2) years (Table 3). Statistical analysis (Kolmogorov test) confirmed significant differences in age distributions between genders (p < 0.001) and the χ2 test revealed associations between hospitalisation rates across different age groups and gender, with a notably higher male-to-female ratio (see S4 Table).

### Analysis of readmitted hospital cases for oral cancer in Hungary (2015–2019)

Between 2015 and 2019, Hungary recorded 25,310 readmitted hospital cases of OC, with a decline from 5,334 cases in 2015–4,763 in 2018, before a minor increase to 4,835 cases in 2019, which also illustrates changes across specific cancer types over time. Table 4 categorises these cases by primary cancer locations and gender distribution, analysing 25,140 cases.

**Table 4:** Main oral cancer locations of hospital readmitted cases and their distributions by genders (percentages as percentages of all readmissions mentioned in the text).

Cancer type	All cases*	Male cases*	Female cases*	Male- per female cases relative frequencies
Malignant neoplasm of other and ill-defined sites in the lip, oral cavity and pharynx	7195 (28.6%)	5469 (21.8%)	1523 (6.1%)	359.1%
Malignant neoplasm of other and unspecified parts of tongue	6147 (24.5%)	4111 (16.4%)	1712 (6.8%)	240.1%
Malignant neoplasm of floor of mouth	4215 (16.8%)	2951 (11.7%)	1006 (4%)	293.3%
Malignant neoplasm of other and unspecified parts of mouth	2067 (8.2%)	1180 (4.7%)	652 (2.6%)	181.0%
Malignant neoplasm of palate	1794 (7.1%)	1134 (4.5%)	606 (2.4%)	187.1%
Malignant neoplasm of gum	1689 (6.7%)	970 (3.9%)	430 (1.7%)	225.6%
Malignant neoplasm of lip	1184 (4.7%)	690 (2.7%)	430 (1.7%)	160.5%
Malignant neoplasm of other and unspecified major salivary glands	849 (3.4%)	356 (1.4%)	364 (1.5%)	97.8%

*Counts and percentages do not add up because Information Governance rules did not allow publish small numbers.

**Fig 8:**
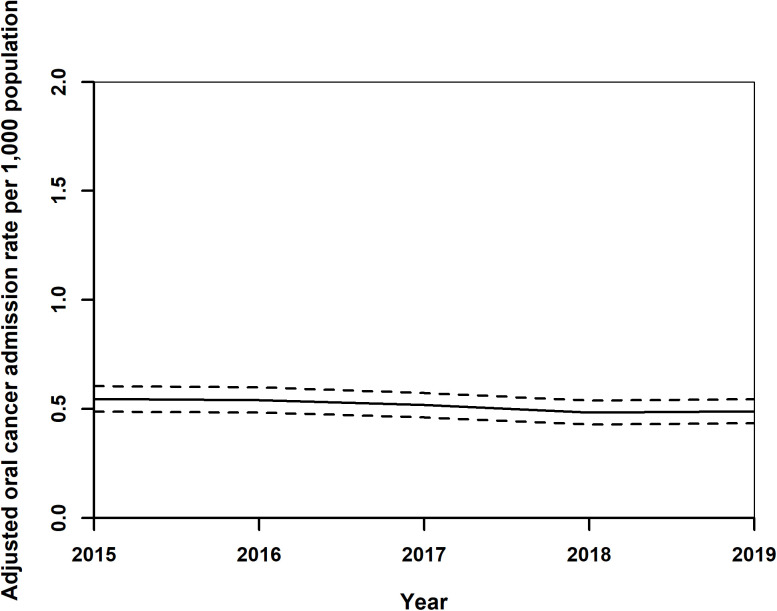
Adjusted standardised oral cancer readmission rates in Hungary from 2015 to 2019.

**Fig 9:**
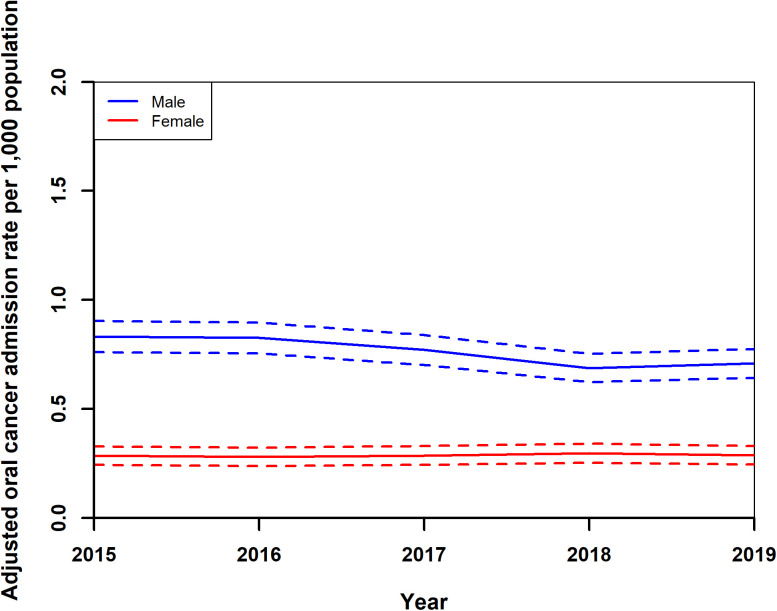
Adjusted standardised oral cancer readmission rates of males and females in Hungary from 2015 to 2019.

**Fig 10:**
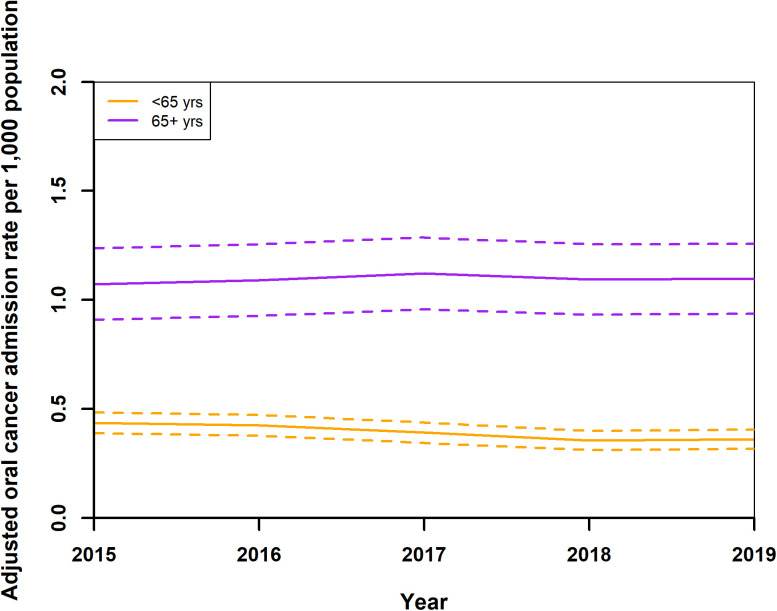
Adjusted standardised oral cancer readmission rates of age group younger than 65 (< 65 yrs)- and older than 65 years (65 ** + yrs) in Hungary from 2015 to 2019**.

**Fig 11:**
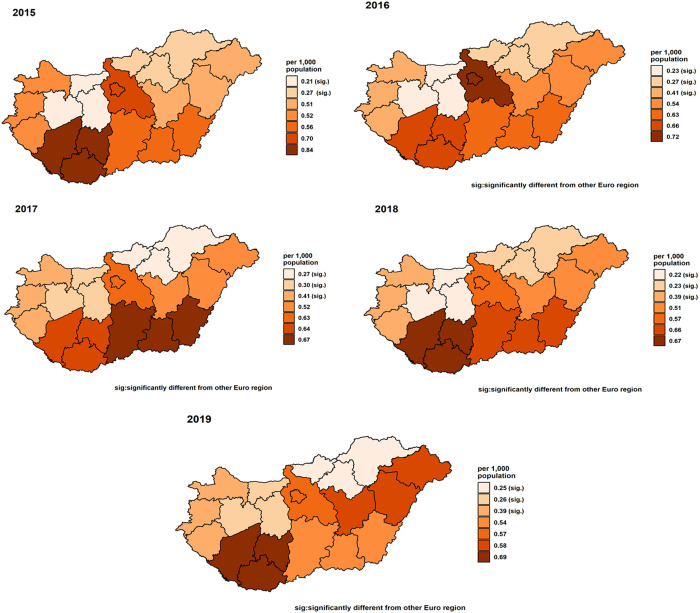
Adjusted standardised oral cancer readmission rates in the seven NUTS 2 regions of Hungary in 2015, 2016, 2017, 2018 and 2019, (different colours depict different NUTS 2 regions).

Standardised readmission rates for OC showed a decrease from 0.55 (0.49,0.61) per 1,000 population in 2015 to 0.37 (0.33,0.42) in 2018, with a slight rise to 0.49 (0.43,0.54) in 2019 (Fig 8). Female rates remained stable around 0.29 (0.24,0.33), while male rates decreased from 0.83 (0.76,0.90) in 2015 to 0.69 (0.62,0.75) in 2018, increasing slightly to 0.71 (0.64,0.78) in 2019 (Fig 9). Adjusted rates for those under 65 years fell from 0.44 (0.39,0.49) to 0.36 (0.32,0.41), and for those over 65, increased from 1.07 (0.91,1.24) to 1.12 (0.96,1.29) in 2017, then declined to 1.10 (0.94,1.26) (Fig 10). After year 2015, regionally, rates generally decreased or did not change, (Fig 11) Central Transdanubia and Northern Hungary observed the lowest rates throughout and the rates of these regions were significantly different from the other regions, it was true about the rate of Western Transdanubia as well.

### Analysis of deaths and comorbidities

#### Mortality and survival in the study population.

Between 2015 and 2019, there were a total of 9,371 deaths among recorded with OC in Hungary, with annual figures increasing from 1,351 in 2015–2,292 in 2019. The age range at death extended from under 2 years to 106 years, with the mean 66.9 (SD: 11.4, median: 66.1) years (Table 5). Detailed gender analysis showed that for males, these were mean 65.6 (SD: 10.7, median: 64.9) years with the youngest under 2 years and the oldest at 101.7 years (Table 5). For females, these figures were slightly higher, with mean 69.9 (SD: 12.4, median:69.6) years and ranging from 4.0 to 106.1 years (Table 5). The analysis revealed a statistically significant difference (p < 0.001) in age distributions between genders.

**Table 5:** Summary statistics (in years) of age of deaths of all-,male- and female cases with oral cancer in Hungary from 2015 to 2019.

	Minimum	Maximum	Mean	Standard deviation	Median
**All cases**	1.8	106.1	66.9	11.4	66.1
**Male**	1.8	101.7	65.6	10.7	64.9
**Female**	4.0	106.1	69.9	12.4	69.5

Additionally, the study found significant associations between gender and geographical locations at the level of counties with mortality number (p < 0.001) (see S5 Table). A characteristic pattern was observed the relation between time-to-death and the time of diagnosis of patients with OC: 46.3% of deaths occurred within a year of an OC diagnosis (Table 6). Subsequent years showed a decrease in this percentage, highlighting the critical time interval of mortality post observation (Table 6). Significant associations were also noted between herein discussed time and gender of patients (p < 0.05), with the male-to-female ratio exceeding 2 within the first two years after diagnosis followed by a decrease in subsequent years (Table 6).

**Table 6:** Time relationship in years between time of death and time of diagnosis of oral cancer in Hungary from 2015 to 2019; of total, male and female death cases with their relevant gender ratios (percentages based on total number of death cases).

Time span between diagnosis and mortality (in years)	Total	Male	Female	Male/female ratio
**<1**	4,335 (46.3%)	2,981 (31.8%)	1,354 (14.5%)	2.20
**1 to <2**	3,043 (32.5%)	2,074 (22.1%)	969 (10.3%)	2.14
**2 to <3**	1,097 (11.7%)	699 (7.5%)	398 (4.3%)	1.76
**3 to <4**	613 (6.5%)	407 (4.3%)	206 (2.2%)	1.97
**4 or more**	283 (3.0%)	183 (1.1%)	100 (1.1%)	1.82

Further analysis of the death cases in the study population showed for the same year death with OC diagnosis that these were generally less than 8%, except of 14.4% and 9.1% for year 2015 and 2019, respectively (Table 7). Percentages of within a year death after OC diagnosis were similar to the same the percentages of same year death except in death year of 2016 where it was 10.7% (Table 7). The within two-, three, four years death after OC diagnosis were in the same magnitude. (Table 7).

**Table 7:** Year of death among patients diagnosed with oral cancer in Hungary from 2015 to 2019 (percentages based on total number death cases).

	Year of death				
Year of oral cancer case diagnosis	2015	2016	2017	2018	2019
**2015**	1,351 (14.4%)	1,002 (10.7%)	502 (5.4%)	398 (4.2%)	283 (3.0%)
**2016**	0 (0.0%)	741 (7.9%)	693 (7.4%)	306 (3.3%)	215 (2.3%)
**2017**	0 (0.0%)	0 (0.0%)	675 (7.2%)	697 (7.4%)	289 (3.1%)
**2018**	0 (0.0%)	0 (0.0%)	0 (0.0%)	713 (7.6%)	651 (6.9%)
**2019**	0 (0.0%)	0 (0.0%)	0 (0.0%)	1 (0.0%)	854 (9.1%)

#### Mortality in control population.

There were altogether 14,221 deaths, namely 2861, 2808, 2875, 2810 and 2867 deaths among the people in the control group in 2015,2016,2017,2018 and 2019, respectively. The age range at death in this group extended from under 1 year to 105.0 years, with mean 74.7 (SD: 11.3, median: 74.4) years. For males, the statistics of this measure was less than 1 year to 104.2 years, with mean 72.4 (SD: 10.8, median: 71.6) years and for females less than 2 years 105.1 years, with mean 78.9 (SD: 11.1, median: 80.8) years. There was a statistically significant difference (p < 0.001) between death age distributions of death of males and females. Associations were found between gender and number of deaths (p < 0.001), death in counties (p < 0.001) and gender, indicating gender and county-level differences in this population (S6 Table).

#### Deaths among hospitalised patients.

During the study period, there were 3,318 deaths among hospitalised OC patients, with annual deaths increasing from 520 in 2015–743 in 2019. Age at death ranged from 9.65 to 99.8 years, with mean 64.6 (SD: 10.4, median: 63.7) years. Male patients had a lower mean age at death (63.4 years) compared to females (68.2 years), indicating a significant gender difference in the pattern of mortality (p < 0.001). Association was found between gender and number of deaths of hospitalised OC patients and (p < 0.001); and county related death of the same patients and gender as well (p < 0.001). This analysis also revealed county-level disparities and a distinct male-to-female death ratio (S7 Table).

#### Comorbidities in the study population.

Table 8 shows the statistics of comorbidities in the study population. In case of each comorbidity variable, the percentage of a relevant comorbidity was higher in the cases group than in the control group. This association was also confirmed by χ2 test for each comorbidity type (p < 0.001). The relative ratios of comorbidities of cases to controls showed the highest values with respect of to the entire spectrum of comorbidities and for alcohol- and respiratory system related comorbidities (Table 8). The percentage of zero or one comorbidity in the control population was higher than in the population affected by OC (Table 9). Percentages of people with at least two comorbidities were significantly higher in the cases group than in the control population group (χ2 test, p < 0.001) (Table 9). The relative ratios of number of comorbidities of cases to controls showed an increasing tendency (Table 8).

**Table 8:** Number and relative risk with 95% confidence interval and p-value of alcohol related, gastrointestinal, stomatological, cardiovascular- and respiratory system related comorbidities in patients with oral cancer patients to control group in Hungary from 2015 to 2019 (percentages as percentages in the relevant population).

Comorbidity	Cases	Control population	Relative risk	p-value
**No comorbidity**	1,840 (8.1%)	23,926 (21.1%)		
**Alcohol related**	1,044 (4.6%)	2,343 (2.1%)	5.79 (5.31,6.33)	<0.001
**Gastro-intestinal**	10,904 (48.1%)	39,554 (34.8%)	3.58 (3.40,3.58)	<0.001
**Stomato-logical**	8971 (39.5%)	31,958 (28.1%)	3.65 (3.46,3.85)	<0.001
**Cardio-vascular**	17,909 (78.9%)	78,473 (69.1%)	2.97 (2.82,3.12)	<0.001
**Respiratory system**	4,641 (20.4%)	11,614 (10.2%)	5.20 (4.90,5.51)	<0.001
**All types of comorbidities**	70 (0.3%)	101 (0.09%)	9.01 (6.52,12.39)	<0.001

**Table 9:** Comorbidity burden in the case and control group of Hungary from 2015 to 2019.

Number of comorbidities	Control population	Cases population	Ratio of the number of comorbidities in the case-to-control population
**0**	23,926 (21.1%)	1,840 (8.1%)	0.4
**1**	36,358 (32.0%)	5,770 (25.4%)	0.8
**2**	34,750 (30.6%)	8,212 (36.2%)	1.2
**3**	15,921 (14.0%)	5,421 (23.9%)	1.7
**4**	2,454 (2.2%)	1,389 (6.1%)	2.8
**5**	101 (0.09%)	70 (0.3%)	3.5
**Total**	113,510 (100%)	22,702 (100%)	

When associations of comorbidities were studied in case- and control groups as related to gender, and age groups of younger than 65 years and older than 65 years, associations were found in both groups with these variables for each specific type of comorbidities and number of comorbidities. Associations were not found neither group between all comorbidities and herein discussed two variables (S8–S11 Tables).

We studied how the number of comorbidities and individual types of comorbidities affect death cases in the study population. The first logistic regression model showed that the odds ratio of death in the population is 5.20 (5.03,5.38) of people with OC compared to people without it (Table 10). Odds ratio of death of females is lower 0.60 (0.58,0.63) than males and death odds ratio of older age group is higher 3.08 (2.98,3.19) than that of the younger one (Table 10). Odds ratios of death with the cumulative number of comorbidities varied between 2.14 (2.07, 2.21) and 2.44 (2.36,2.52) and increased the odds ratio of death as compared to patients with OC but without any comorbidity (Table 10). By an extended model, interaction was found (p < 0.001) between at least one, maximum three comorbidities and the older than 65 years of age group.

**Table 10:** Odds ratios of death in the Hungarian sample population between 2015 and 2019 by multiple logistic regression model with demographic variables and number of comorbidities.

	Odd ratio (95% confidence interval)	p-value
**Oral cancer case:**		
** Yes/No**	5.20 (5.03,5.38)	<0.001
**Gender:**		
** Female/Male**	0.60 (0.58,0.63)	<0.001
**Age group:**		
** Over 65 years/Under 65 years**	3.08 (2.98,3.19)	<0.001
**Number of comorbidities:**		
** 1/None**	2.32 (2.24,2.40)	<0.001
** 2/None**	2.20 (2.13,2.28)	<0.001
** 3/None**	2.14 (2.07,2.21)	<0.001
** 4/None**	2.16 (2.09,2.24)	<0.001
** 5/None**	2.44 (2.36,2.52)	<0.001

Using another logistic regression model when specific comorbidities were taken into account, we confirmed the significant effect of (i) presence of OC vs. no OC, (ii) the female gender, and (iii) the older than 65 years of age group on odds ratios of death with similar values 5.84 (5.64,6.04), 0.62 (0.60,0.64) and 2.63 (2.55,2.73), as estimated by the previous model (Tables 10, 11). Considering specific types of comorbidities: existence of alcohol related, gastrointestinal, cardiovascular- and respiratory system related comorbidities with 2.73 (2.64,2.82), 1.07 (1.04,1.11), 1.87 (1.81,1.94) and 2.32 (2.16,2.31), respectively increased death odds ratios in the study population (Table 11). It was found that stomatological comorbidity decreased death odds ratio 0.32 (0.31,.034) in the population (Table 11).

**Table 11:** Odds ratios of death in the Hungarian sample population between 2015 and 2019 by multiple logistic regression model with demographic variables and specific types comorbidities.

	Odd ratio (95% confidence interval)	p-value
**Oral cancer case:**		
** Yes/No**	5.84 (5.64,6.04)	<0.001
**Gender:**		
** Female/Male**	0.62 (0.60,0.64)	<0.001
**Age groups:**		
** Over 65 years/ Under 65 years**	2.63 (2.55,2.73)	<0.001
**Type of comorbidity:**		
** Alcohol related comorbidities**		
** Yes/No**	2.73 (2.64,2.82)	<0.001
** Stomatological comorbidities**		
** Yes/No**	0.32 (0.31,0.34)	<0.001
** Gastrointestinal comorbidities**		
** Yes/No**	1.07 (1.04,1.11)	<0.001
** Cardiovascular comorbidities**		
** Yes/No**	1.87 (1.81,1.94)	<0.001
** Respiratory system comorbidities**		
** Yes/No**	2.23 (2.16,2.31)	<0.001

#### Comorbidities of hospitalised patients.

Of the 6,356 hospitalised patients with OC, comorbidities were distributed as follows: alcohol related disorders (307, 4.8%), diseases of the gastrointestinal system (3,054, 48.1%), stomatological disorders (3,003, 47.3%), cardiovascular disorders (4,904, 77.2%), and diseases of the respiratory system (1,181, 18.6%). Nineteen 19 (0.3%) patients presented all five comorbidities (Table 12). A subset of 580 (9.1%) patients had no comorbidities. The number and percentage of comorbidities among hospitalised patients were: zero (580, 9.1%), one (1,554, 24.5%), two (2,228, 35.1%), three (1,556, 24.5%), four (419, 6.6%), and all five (19, 0.3%). Significant associations were observed between the number of comorbidities and gender (p < 0.001), with specific associations between comorbidity counts and age groups under and over 65 years (p < 0.001), as well as mortality (p < 0.001), except for alcohol-related and respiratory system comorbidities which showed no statistically significant associations (p = 0.20 and p = 0.27, respectively).

**Table 12:** Types of comorbidities, relative risks with their confidence intervals and p-values among patients hospitalised with oral cancer in Hungary from 2015 to 2019 (percentages as percentages of all hospitalised patient).

Type of comorbidity	Yes	No	Relative risk	p-value
**No comorbidity**	580 (9.1%)	5776 (90.9%)		
**Alcohol related**	307 (4.8%)	6049 (95.2%)	0.51 (0.44,0.58)	<0.001
**Gastro intestinal**	3,054 (48.1%)	3,302 (51.9%)	9.21 (8.34,10.18)	<0.001
**Stomatological**	3,003 (47.3%)	3,353 (52.7%)	8.92 (8.07,9.86)	<0.001
**Cardiovascular system**	4,904 (77.2%)	1,452 (22.8%)	33.62 (30.30,37.33)	<0.001
**Respiratory system**	1,181 (18.6%)	5,175 (81.4%)	2.72 (2.04,2.53)	<0.001
**All comorbidities**	19 (0.3%)	6,337 (99.7%)	0.03 (0.02,0.05)	<0.001

## Discussion

When cancer cases of this study were compared to the publicly available similar data of the Hungarian Cancer Registry [7], the number of cases in our data was 3–5 times than the latter ones. This difference might be due to the fact that the registry data are being reported by Hungarian hospitals [6] while claimed data were used in this study and the fact that we used administrative data compared to a patient registry. The difference lies in the method: claim data contain all record even with a possible OC diagnosis. Registries should only cover patient with proven OC diagnosis. When the first admissions of our hospital data were compared with the herein mentioned registry data, a very good agreement was found.

Variant Coronavirus 2 (CoV-2)-related COVID-19 pandemic profoundly affected patients with oral- and oropharyngeal cancers worldwide [15]. This study used data before the pandemic therefore, it shows an accurate picture on the affected population.

### Incidence, mortality and demographic trends

In the current study, gender distribution showed a predominance of male cases, with males having nearly 1.5 times more cases than females. This gender disparity is similar to global trends according to the Global Burden of Disease (GBD) study [16], where males consistently show higher ASIR compared to females. In 2019, the global ASIR for males was 6.16 per 100,000, while for females it was 3.01 per 100,000 [2,17]. This disparity could be attributed to the fact that males are more likely to engage in risk behaviours such as tobacco and alcohol use with a higher prevalence of heavy smoking and binge drinking [18,19], which are major risk factors for OC. Occupational exposures to carcinogens are also more common in industries dominated by male workers [20]. Studies by Davis JT et al. [21] indicated that men were less willing to participate in cancer screening programs compared to women, contributing to later-stage diagnoses and higher incidence rates. Additionally, a national survey by the Cleveland Clinic revealed that many men refused going to medical examinations and hold back on certain issues once they were at the doctor’s office, which could lead to delayed diagnosis and less effective treatment at a later stage [22]. Another study by Sfeatcu R et al. [23] highlighted that women had more positive attitudes towards attending dental and medical services, seeking higher oral health values, exhibiting better self-care behaviours, and higher oral health literacy than men.

In Hungary, ASIR for both genders initially showed a moderate decrease from 2016 to 2018, followed by a slight increase in 2019. These trends in the Hungarian ASIR values show some similarity to these noted in Western Europe, where the ASIR remained stable for females, around 3.73 to 3.70, and showed a slight decrease for males, from 8.65 to 8.40 [16]. In contrast, in Central Europe, the ASIR for males remained relatively stable, around 9.30 in 2016 to 9.38 in 2019, while females experienced a slight increase from 2.53 to 2.64. In Eastern Europe, there was a noticeable increase in ASIR for both genders, with males increasing from 10.56 to 11.51 and females from 2.62 to 3.00.

Key interventions include the “Public Health Products Tax,” which was introduced in 2011, aimed at reducing unhealthy product consumption, and stricter tobacco control measures, such as higher taxes, smoking bans, graphic health warnings, and plain packaging, which contributed to a decline in smoking prevalence, as confirmed by WHO and IndexMundi data [24–27]. Additionally, increased alcohol taxes and public awareness campaigns targeted alcohol consumption [28]. The expansion of the National Cancer Screening Program and school-based health education further supported early detection and healthier behaviours, collectively contributing to the observed decline in OC incidence [29]. An interesting factor to consider for future trends is the impact of HPV vaccination. Although the vaccinated population primarily consists of school-aged children, which does not directly represent the older adult patient population addressed in the current study, the high uptake rate of 80% among teenagers in 2019, as reported by the Hungarian Medical Association, may contribute to future declines in OC incidence [30]. HPV is a known risk factor for OCs, as highlighted by a meta-analysis by Haghshenas MR et al., which found an odds ratio of 4.00 (95% CI: 2.31, 6.93) for HPV infection and OC risk [31].

In parallel with the previously discussed overall and gender-specific trends, age-specific trends in Hungary demonstrated a modest decrease in ASIR for both individuals under 65 and those 65 and older from 2016 to 2018, followed by a slight increase in 2019. The adjusted standardised rates for individuals under 65 showed a decrease from 2016 to 2018, then slightly increased in 2019. For those 65 and older, the rates also decreased over the same period of time, with a minor increase in 2019. This pattern contrasts with broader European trends, where ASIR generally increased in Eastern Europe, remained stable in Western Europe, and showed minor fluctuations in Central Europe. The explanations for Hungary’s trends apply here as well, highlighting the impact of sustained public health efforts on reducing OC incidence.

In terms of mortality due to OCs, the current study found that males had higher mortality rates and younger ages at death compared to females, consistent with GBD data, which shows higher mortality rates for males globally and across European regions. From 2016 to 2019, global mortality rates slightly increased for both genders. In Eastern Europe, mortality rates remained stable for males but slightly increased for females. In Central Europe, mortality rates increased for both genders, while in Western Europe, rates were stable for males and slightly increased for females.

The persistently higher ASIR in Western Transdanubia, significantly above all other NUTS 2 regions from 2017 onward, may reflect both true excess risk and more intensive case detection. Eurostat’s 2023 Regional Yearbook reports that Western Transdanubia ranks in the top quartile nationally for hospital‐bed and specialist‐physician density [32], while the Hungarian Central Statistical Office data indicate that per‐capita alcohol consumption and smoking prevalence here exceed the Hungarian average [33]. In contrast, Northern Hungary and Central Transdanubia, which show lower incidence and readmission rates, are among the regions with the scarcest oncology services and lower preventive‐care uptake, raising the possibility of under‐ascertainment or delayed diagnosis.

Despite a decrease in OC incidence in Hungary, mortality rates are still high and show rising trends, when compared to the Central European age-standardised cause-specific mortality data. This can be attributed to several factors. There is a shortage of oncology specialists in regional and county centres, with the National Institute of Oncology, among a few other centres, having enough qualified oncologists, radiotherapists, radiologists, and residents. Additionally, Hungary’s GDP-based national budget for health in general and in particular for cancer care falls short of EU standards. In 2018, Hungary spent €226 per person on cancer patients, only 69 percent of the EU average of €326. Total spending on cancer was estimated at €1,372 million, with nearly half spent on cancer care and two-thirds of that on cancer drugs. This financial disparity, combined with less effective treatment and late-stage diagnosis, underscores the urgent need to improve the quality of cancer care in Hungary, as highlighted by the OECD’s Cancer Profile [34,35].

### Tumor sites and comorbidities

The study identified the tongue, the floor of the mouth, and other ill-defined sites in the lip, oral cavity, and pharynx as the most common tumour sites, with a marked male predominance. This finding is similar to the results of other studies. Dhanuthai K et al. reported the tongue (25.4%), labial/buccal mucosa (21.7%), and gingiva (14.0%) as the primary sites, with a male-to-female ratio of 2.22:1 [36]. Similarly, Al-Rawi NH et al. identified the tongue (57.6%), cheeks (28.1%), and jaw bones (10.8%) as common sites, also showing a male predominance with a ratio of 2.5:1 [37]. O Stepan K et al. noted the tongue (41.7%) and floor of mouth (16.5%) as the most frequent sites [38]. All these studies, like the present one, demonstrated a higher incidence of OC in males, emphasizing the consistent gender disparities. This high prevalence in the tongue and floor of the mouth can be attributed to several factors. These areas have a rich blood and lymphatic vessel supply, facilitating the spread and growth of cancer cells [39]. These sites are directly exposed to carcinogens from tobacco and alcohol consumption, substances more frequently used by males [40]. Regular mechanical irritation from dental appliances, sharp teeth, or rough dental fillings can cause chronic trauma to the mucosa, increasing cancer risk [41]. HPV infection is another contributing factor, particularly affecting the oropharyngeal region [42]. Additionally, saliva pooling in the floor of the mouth can harbour carcinogens for prolonged periods. The thin, non-keratinized epithelium in these areas, combined with high cell turnover rates, makes them more susceptible to malignant transformation [43].

The study revealed that hospitalised OC patients had significantly higher rates of all comorbidity types, including alcohol-related, gastrointestinal, stomatologic, cardiovascular, and respiratory system conditions, compared to the control population. This elevated comorbidity burden is likely exacerbated by shared risk factors such as tobacco smoking and alcohol consumption, which are known to contribute to both OC and these comorbid conditions. For instance, smoking and alcohol consumption are major risk factors for hypertension, ischemic heart disease, peripheral artery disease, gastric ulcers, chronic obstructive pulmonary disease (COPD), and periodontal disease [44–46]. Additionally, the number of comorbidities was notably higher in the case group, emphasizing the compounded health challenges faced by OC patients. These findings align with those of Ghanizada M et al. and Yang YH et al., who also reported significant comorbidities in their OC patient cohorts [47,48].

The high comorbidity burden among OC patients is a significant concern. Only a small percentage of these patients have no comorbidities, and many have multiple comorbid conditions, reflecting trends observed in broader cancer populations. Chien LH et al. noted that in Taiwan, comorbidities in patients with common cancers, including OC, correlated with noncancer-related deaths, highlighting the critical impact of these additional health issues [49]. Similarly, Fowler H et al. found a high prevalence of comorbidities among cancer patients, with many of them having multiple conditions [50].

Patients with OC present with more comorbidities than the control population due to the systemic effects of their risk factors and the disease itself. The carcinogenic effects of tobacco and alcohol, common risk factors for OC, lead to physiological damage, causing chronic conditions such as cardiovascular and respiratory diseases [51]. Moreover, the immunosuppressive effects of OC and its treatments further exacerbate existing conditions and increase susceptibility to new ones [52,53]. Addressing these comorbidities concurrently with cancer treatment is crucial for improving patient outcomes. Aksoy S et al. highlighted that managing comorbid conditions alongside cancer treatment can significantly enhance patient prognosis and quality of life [54].

### Impact of comorbidities on mortality of OC patients

The study demonstrated that the presence and number of comorbidities significantly increased the risk of mortality in patients with OC, with a stepwise increase in odds ratios with each additional comorbidity according to results of the logistic regression model. This finding is consistent with research by Davies L et al., which showed that patients with OC have worse other-cause survival compared to the general population, with a higher risk of death from noncancer causes even at localized stages. The risk of death from other causes increases further at advanced cancer stages [55,56].

Søgaard M et al. conducted an extensive review of over 2,500 articles related to comorbidity and cancer [57]. They found that cancer patients with comorbidities generally have poorer survival rates, with 5-year mortality hazard ratios ranging from 1.1 to 5.8. The presence of severe comorbidities or psychiatric disorders was associated with delayed cancer diagnosis, while chronic diseases requiring regular medical visits were linked to earlier cancer detection.

Moreover, they noted that patients with comorbidities often receive less standard cancer treatment, such as surgery, chemotherapy, and radiation therapy, and have lower completion rates for these treatments [57]. The reasons for this may include considerations of greater toxicity risk, poorer general health performance status, patients’ preferences, and poor adherence among patients with comorbidities. Results of the present study were supported by recent findings indicating that with the increase in comorbidity levels, delays in diagnosis and treatment completion become more pronounced, particularly in patients over 65 years. This leads to a significantly higher risk of mortality [58].

The analysis of specific comorbidities provides further understanding of their impact on the outcome of OC. In line with the findings of the present study, another study demonstrated that hypertension and cardiac diseases, including congestive heart failure and myocardial infarction, were prevalent and associated with significant long-term health risks [59]. The results of the logistic regression model in the current study’s results indicated that cardiovascular comorbidities substantially increased the risk of mortality, which is consistent with the observed high cumulative probabilities of these conditions. Similarly, the significant presence of COPD and pneumonia in the other study [60] aligns with the current finding that respiratory system-related comorbidities markedly elevate the risk of death. The study also highlighted the prevalence of hyperlipidaemia and diabetes, conditions which the current results suggested to contribute to increased mortality risk. Interestingly, while stomatologic comorbidities were less prevalent in the other study, the current findings indicate a protective effect, potentially due to an indicator role for revealing OC at early stages.

Cardiovascular disease exerts its deleterious effect in oral cancer patients through several interrelated pathways. Chronic endothelial dysfunction and systemic inflammation, hallmarks of hypertension and atherosclerosis, can impair microvascular perfusion within the tumor microenvironment, reducing oxygen delivery and hampering the efficacy of radiotherapy and chemotherapy. Moreover, pre-existing cardiac dysfunction limits patients’ ability to tolerate aggressive multimodal treatment, leading to dose reductions or treatment delays that compromise tumor control [61]. Gastrointestinal comorbidities, present in nearly half of our cohort, principally drive malnutrition and cachexia: impaired nutrient intake and absorption precipitate weight loss and hypoalbuminemia, which weaken immune competence and delay wound healing after surgery or mucosal injury from chemoradiation [62]. In contrast, the apparent “protective” association of stomatological comorbidities likely reflects the impact of regular dental surveillance: patients engaged in routine oral health care are more apt to have premalignant lesions biopsied early, resulting in earlier‐stage diagnoses and improved outcomes [63].

The study revealed that the majority of deaths occurred within one year diagnosis. Cancer Research UK data show that over 75% of patients with OC survive at least one year after diagnosis [64,65]. The NORDCAN database reported one-year survival rates for male patients with OC ranging from 56.3% in Denmark to 65.2% in Norway, and for female OC from 64.4% in Denmark to 74.2% in Finland [66]. These findings highlight the particularly severe prognosis of OC in the present Hungarian study cohort. One of the factors contributing to this low one-year survival in Hungary is the significant patient delay, with a median of 9.5 weeks and an average of 17.57 weeks from the onset of symptoms to the first medical consultation, when many patients presented with a locoregionally advanced disease [67]. This delay is longer compared to other countries and is a crucial factor contributing to high mortality. According to Pozsgai E et al. not only OC but a significant proportion of total cancer cases in Hungary were diagnosed at advanced stages [68].

### Strengths and limitations

The strength of this study is that it used real-world, population-based administrative data with respect to case numbers, incidence- and hospitalisation rates as well as comorbidities. For estimating standardised rates, yearly Hungarian population-wide data were used. Matched case-control data were used resulting robust results of logistic regressions analyses. Impact of comorbidities both by disease types, and their cumulative number was also evaluated. We were able to show geographical differences, which is also a novelty of this study. One of the limitations of our approach was that data were provided for incidence cases only from the year of 2016; therefore, incidence-related calculations resulted in a delay of one year from the start of the study. The few data points of incidence figures, i.e., four ones from 2016 to 2019, did not allow a quantitative trend analysis using statistical methods. A further limitation of this study was that causes of death were not available in the database used, therefore, we could discuss mortality of Hungarian patients with OC in general in general terms. Also the relatively short time frame limits our ability to assess long-latency effects characteristic of oral cancer. Tobacco use information was not recorded and could only be inferred indirectly via associated diseases, and causes of death were not captured in the NHIF database, restricting our mortality discussion to overall survival rather than cancer-specific outcomes.

## Conclusion

While our data from 2016 to 2019 hint at a modest decline in Hungary’s oral cancer incidence, potentially reflecting recent population-level preventive measures, this short observation period precludes firm conclusions, and ongoing surveillance with additional years of data will be essential to determine whether these early signals represent a sustained trend. Significant challenges still remain in reducing mortality rates and improving survival especially within the first year from diagnosis. There is a critical need to enhance screening programs for early detection, enabling treatment at less advanced stages where interventions can be more effective. Implementing a risk-based approach for older patients and focusing on proper parallel management of coinciding comorbid conditions represent essential strategies to improve outcomes and reduce mortality. Future research should utilise non-aggregated data and a longitudinal design to better assess causal and temporal relationships characterising the epidemiology of OC in Hungary.

## Supporting information

S1 Table ICD-10 codes defines the oral cancer in the sudy. (DOCX)

S2 Table ICD-10 codes describe the comorbidities of oral cancers in Hungary from 2015 to 2019. (DOCX)

S3 Table Number of all, male- and female patients in different age groups with oral cancer in Hungary from 2015 to 2019. (DOCX)

S4 Table Number of all-, male- and female patients, gender ratios in different age groups hospitalised with oral cancer in Hungary from 2015 to 2019. (DOCX)

S5 Table Number of male- and female deaths, and their ratio among oral cancer cases in the different counties of Hungary from 2015 to 2019. (DOCX)

S6 Table Number of male- and female deaths, and their ratio among control population of oral cancer cases in the different counties of Hungary from 2015 to 2019. (DOCX)

S7 Table Number of male- and female deaths, and their ratio among hospitalised oral cancer patients in the different counties of Hungary from 2015 to 2019. (DOCX)

S8 Table Different types of comorbidities in the case and control group of Hungary from 2015 to 2019 in different genders (percentages as percentages in the relevant population). (DOCX)

S9 Table Different types of comorbidities in the case and control group of Hungary from 2015 to 2019 in different age groups less than 65 years (<65 years) and equal or large than 65 years (65 + years) (percentages as percentages in the relevant population). (DOCX)

S10 Table Comorbidity burden in the case and control group of Hungary from 2015 to 2019 in different genders (percentages as percentages in the relevant population). (DOCX)

S11 Table Comorbidity burden in the case and control group of Hungary from 2015 to 2019 in different age groups less than 65 years (<65 years) and equal or large than 65 years (65+ years) (percentages as percentages in the relevant population). (DOCX)
