# Supplementary material for: Oral cancer in Hungary: An epidemiological profile (2015–2019)
Source: PLoS One. 2025 Jul 3;20(7):e0327566. doi: 10.1371/journal.pone.0327566 (PMC12225832; doi:10.1371/journal.pone.0327566)
Supplement: S1 Table — (DOCX) [file pone.0327566.s001.docx]

**S1 Table: ICD-10 codes defines the oral cancer in the sudy.**

| **Cancer Type** | **Description** |
| --- | --- |
| C00 | Malignant neoplasm of lip |
| C02 | Malignant neoplasm of other and unspecified parts of tongue |
| C03 | Malignant neoplasm of gum |
| C04 | Malignant neoplasm of floor of mouth |
| C05 | Malignant neoplasm of palate |
| C06 | Malignant neoplasm of other and unspecified parts of mouth |
| C08 | Malignant neoplasm of other and unspecified major salivary glands |
| C14 | Malignant neoplasm of other and ill-defined sites in the lip, oral cavity and pharynx |
