# Supplementary material for: Oral cancer in Hungary: An epidemiological profile (2015–2019)
Source: PLoS One. 2025 Jul 3;20(7):e0327566. doi: 10.1371/journal.pone.0327566 (PMC12225832; doi:10.1371/journal.pone.0327566)
Supplement: S2 Table — (DOCX) [file pone.0327566.s002.docx]

**S2 Table: ICD-10 codes describe the comorbidities of oral cancers in Hungary from 2015 to 2019.**

| **Alcohol related medical conditions** | **Description** |
| --- | --- |
| K70 | Alcoholic liver disease |
| K72 | Hepatic failure, not elsewhere classified |
| K74 | Fibrosis and cirrhosis of liver |
| K86 | Other diseases of pancreas |
| **Gastrointestinal medical conditions** |  |
| K21 | Gastro-oesophageal reflux disease |
| K22 | Other diseases of oesophagus |
| K25 | Gastric ulcer |
| K26 | Duodenal ulcer |
| K29 | Gastritis and duodenitis |
| K66 | Other disorders of peritoneum |
| **Cardiovascular conditions** |  |
| I10 | Essential (primary) hypertension |
| I11 | Hypertensive heart disease |
| I13 | Hypertensive heart and renal disease |
| I20 | Angina pectoris |
| I15 | Secondary hypertension |
| I21 | Acute myocardial infarction |
| I22 | Subsequent myocardial infarction |
| I23 | Certain current complications following acute myocardial infarction |
| I24 | Other acute ischaemic heart diseases |
| I25 | Chronic ischaemic heart disease |
| I42 | Cardiomyopathy |
| I51 | Complications and ill-defined descriptions of heart disease |
| I61 | Intracerebral haemorrhage |
| I62 | Other nontraumatic intracranial haemorrhage |
| I63 | Cerebral infarction |
| I64 | Stroke, not specified as haemorrhage or infarction |
| I67 | Other cerebrovascular diseases |
| I69 | Sequelae of cerebrovascular disease |
| I70 | Atherosclerosis |
| I73 | Other peripheral vascular diseases |
| I77 | Other disorders of arteries and arterioles |
| I83 | Varicose veins of lower extremities |
| I85 | Oesophageal varices |
| I98 | Other disorders of circulatory system in diseases classified elsewhere |
| **Respiratory system related medical conditions** |  |
| J44 | Other chronic obstructive pulmonary disease |
| J96 | Respiratory failure, not elsewhere classified |
| **Stomatological conditions** |  |
| K00 | Disorders of tooth development and eruption |
| K02 | Dental caries |
| K03 | Other diseases of hard tissues of teeth |
| K05 | Gingivitis and periodontal diseases |
| K08 | Other disorders of teeth and supporting structures |
| K11 | Diseases of salivary glands |
| K14 | Diseases of tongue |
