# Supplementary material for: Oral cancer in Hungary: An epidemiological profile (2015–2019)
Source: PLoS One. 2025 Jul 3;20(7):e0327566. doi: 10.1371/journal.pone.0327566 (PMC12225832; doi:10.1371/journal.pone.0327566)
Supplement: S3 Table — (DOCX) [file pone.0327566.s003.docx]

**S3 Table: Number of all, male- and female patients in different age groups with oral cancer in Hungary from 2015 to 2019.**

|  | **< 25**  **years** | **25-<35 years** | **35-<45 years** | **45-<55 years** | **55-<65 years** | **65-<75**  **years** | **75+**  **years** |
| --- | --- | --- | --- | --- | --- | --- | --- |
| All | 562 | 485 | 1161 | 2876 | 6841 | 6355 | 4422 |
| Male | 283 | 213 | 533 | 1855 | 4486 | 3843 | 2124 |
| Female | 279 | 272 | 628 | 1021 | 2355 | 2512 | 2298 |
| Male and female ratio | 1.01 | 0.78 | 0.85 | 1.82 | 1.90 | 1.53 | 0.92 |
