# Supplementary material for: Oral cancer in Hungary: An epidemiological profile (2015–2019)
Source: PLoS One. 2025 Jul 3;20(7):e0327566. doi: 10.1371/journal.pone.0327566 (PMC12225832; doi:10.1371/journal.pone.0327566)
Supplement: S4 Table — (DOCX) [file pone.0327566.s004.docx]

**S4 Table: Number of all-, male- and female patients, gender ratios in different age groups hospitalised with oral cancer in Hungary from 2015 to 2019.**

|  | **< 25**  **years** | **25-<35 years** | **35-<45 years** | **45-<55 years** | **55-<65 years** | **65-<75**  **years** | **75+**  **years** |
| --- | --- | --- | --- | --- | --- | --- | --- |
| All | 25 | 39 | 212 | 994 | 2434 | 1700 | 952 |
| Male | 15 | 21 | 136 | 782 | 1797 | 1176 | 485 |
| Female | 10 | 18 | 76 | 212 | 637 | 524 | 467 |
| Male and female ratio | 1.50 | 1.17 | 1.79 | 3.69 | 2.82 | 2.24 | 1.04 |
