# Supplementary material for: Oral cancer in Hungary: An epidemiological profile (2015–2019)
Source: PLoS One. 2025 Jul 3;20(7):e0327566. doi: 10.1371/journal.pone.0327566 (PMC12225832; doi:10.1371/journal.pone.0327566)
Supplement: S5 Table — (DOCX) [file pone.0327566.s005.docx]

**S5 Table: Number of male- and female deaths, and their ratio among oral cancer cases in the different counties of Hungary from 2015 to 2019.**

| **County name** | **Male** | **Female** | **Male/Female ratio** |
| --- | --- | --- | --- |
| Baranya | 231 | 85 | 2.72 |
| Bács-Kiskun | 397 | 168 | 2.36 |
| Békés | 201 | 104 | 1.93 |
| Borsod-Abaúj-Zemplén | 423 | 181 | 2.34 |
| Csongrád | 230 | 108 | 2.13 |
| Fejér | 288 | 115 | 2.50 |
| Győr-Moson-Sopron | 233 | 110 | 2.12 |
| Hajdú-Bihar | 348 | 132 | 2.64 |
| Heves | 180 | 72 | 2.50 |
| Komárom-Esztergom | 189 | 87 | 2.17 |
| Nógrád | 182 | 104 | 1.75 |
| Pest | 816 | 428 | 1.91 |
| Somogy | 208 | 97 | 2.14 |
| Szabolcs-Szatmár-Bereg | 315 | 131 | 2.4 |
| Szolnok | 269 | 95 | 2.83 |
| Tolna | 213 | 78 | 2.73 |
| Vas | 155 | 60 | 2.58 |
| Veszprém | 309 | 136 | 2.27 |
| Zala | 171 | 78 | 2.19 |
| Budapest | 886 | 640 | 1.38 |
| Missing* | 100 | 18 | 5.56 |

*: There was no related postcode
