# Supplementary material for: Oral cancer in Hungary: An epidemiological profile (2015–2019)
Source: PLoS One. 2025 Jul 3;20(7):e0327566. doi: 10.1371/journal.pone.0327566 (PMC12225832; doi:10.1371/journal.pone.0327566)
Supplement: S6 Table — (DOCX) [file pone.0327566.s006.docx]

**S6 Table: Number of male- and female deaths, and their ratio among control population of oral cancer cases in the different counties of Hungary from 2015 to 2019.**

| **County name** | **Male** | **Female** | **Male/Female ratio** |
| --- | --- | --- | --- |
| Baranya | 295 | 152 | 1.94 |
| Bács-Kiskun | 561 | 288 | 1.95 |
| Békés | 335 | 168 | 1.99 |
| Borsod-Abaúj-Zemplén | 642 | 312 | 2.06 |
| Csongrád | 307 | 142 | 2.16 |
| Fejér | 399 | 177 | 2.25 |
| Győr-Moson-Sopron | 341 | 167 | 2.04 |
| Hajdú-Bihar | 510 | 243 | 2.1 |
| Heves | 289 | 199 | 1.45 |
| Komárom-Esztergom | 282 | 148 | 1.91 |
| Nógrád | 262 | 150 | 1.75 |
| Pest | 1082 | 650 | 1.66 |
| Somogy | 281 | 144 | 1.95 |
| Szabolcs-Szatmár-Bereg | 441 | 194 | 2.27 |
| Szolnok | 396 | 172 | 2.30 |
| Tolna | 302 | 127 | 2.38 |
| Vas | 238 | 96 | 2.48 |
| Veszprém | 417 | 251 | 1.66 |
| Zala | 411 | 240 | 1.71 |
| Budapest | 1326 | 1028 | 1.29 |
| Missing* | 42 | 14 | 3.00 |

*: There was no related postcode
