# Supplementary material for: Oral cancer in Hungary: An epidemiological profile (2015–2019)
Source: PLoS One. 2025 Jul 3;20(7):e0327566. doi: 10.1371/journal.pone.0327566 (PMC12225832; doi:10.1371/journal.pone.0327566)
Supplement: S7 Table — (DOCX) [file pone.0327566.s007.docx]

**S7 Table: Number of male- and female deaths, and their ratio among hospitalised oral cancer patients in the different counties of Hungary from 2015 to 2019.**

| **County name** | **Male** | **Female** | **Male/Female ratio** |
| --- | --- | --- | --- |
| Baranya | 169 | 52 | 3.25 |
| Bács-Kiskun | 182 | 45 | 4.04 |
| Békés | 84 | 20 | 4.2 |
| Borsod-Abaúj-Zemplén | 160 | 48 | 3.33 |
| Csongrád | 144 | 45 | 3.20 |
| Fejér | 72 | 16 | 4.50 |
| Győr-Moson-Sopron | 78 | 25 | 3.12 |
| Hajdú-Bihar | 213 | 69 | 3.09 |
| Heves | <10* | <10* |  |
| Komárom-Esztergom | 32 | 14 | 2.29 |
| Nógrád | <10* | <10* |  |
| Pest | 47 | 27 | 1.74 |
| Somogy | 112 | 28 | 4.00 |
| Szabolcs-Szatmár-Bereg | 112 | 27 | 4.15 |
| Szolnok | 71 | 22 | 3.23 |
| Tolna | <10* | <10* |  |
| Vas | 100 | 26 | 3.85 |
| Veszprém | 101 | 36 | 2.81 |
| Zala | <10* | <10* |  |
| Budapest | 676 | 302 | 2.24 |

<10*: Information Governance Rules did not allow publish these numbers
