# Supplementary material for: Oral cancer in Hungary: An epidemiological profile (2015–2019)
Source: PLoS One. 2025 Jul 3;20(7):e0327566. doi: 10.1371/journal.pone.0327566 (PMC12225832; doi:10.1371/journal.pone.0327566)
Supplement: S8 Table — (DOCX) [file pone.0327566.s008.docx]

**S8 Table. Different types of comorbidities in the case and control group of Hungary from 2015 to 2019 in different genders (percentages as percentages in the relevant population).**

|  | **Control population** | | | **Cases population** | | |
| --- | --- | --- | --- | --- | --- | --- |
| **Types of comorbidities** | **Male** | **Female** | **p-value** | **Male** | **Female** | **p-value** |
| **Alcohol related** | 1,592 (1.4%) | 751 (0.7%) | <0.001 | 695 (3.1%) | 349 (1.5%) | <0.001 |
| **Gastro intestinal** | 21,586 (19%) | 17,968 (15.8%) | <0.001 | 6,607 (29.1%) | 5,191 (22.9%) | <0.001 |
| **Stomatological** | 17,927 (15.8%) | 14,031 (12.4%) | <0.001 | 51,90 (22.9%) | 3,781 (16.7%) | <0.001 |
| **Cardiovascular system** | 45,486 (40.1%) | 32,987 (29.1%) | <0.001 | 10,270 (45.2%) | 7,639 (33.6%) | <0.001 |
| **Respiratory system** | 7,121 (6.3%) | 4,493 (4%) | <0.001 | 3,067 (13.5%) | 1,726 (7.6%) | <0.001 |
| **All comorbidities** | 63 (0.1%) | 38 (0%) | 0.52 | 44 (0.2%) | 26 (0.1%) | 0.56 |
