# Supplementary material for: Oral cancer in Hungary: An epidemiological profile (2015–2019)
Source: PLoS One. 2025 Jul 3;20(7):e0327566. doi: 10.1371/journal.pone.0327566 (PMC12225832; doi:10.1371/journal.pone.0327566)
Supplement: S9 Table — (DOCX) [file pone.0327566.s009.docx]

**S9 Table. Different types of comorbidities in the case and control group of Hungary from 2015 to 2019 in different age groups less than 65 years (<65 years) and equal or large than 65 years (65+ years) (percentages as percentages in the relevant population).**

|  | **Control population** | | | **Cases population** | | |
| --- | --- | --- | --- | --- | --- | --- |
| **Types of comorbidities** | **<65 years** | **65+ years** | **p-value** | **<65 years** | **65+ years** | **p-value** |
| **Alcohol related** | 1,251 (1.1%) | 1,092 (1%) | <0.001 | 668 (2.9%) | 376 (1.7%) | <0.001 |
| **Gastro intestinal** | 19,538 (17.2%) | 20,016 (17.6%) | <0.001 | 6,235 (27.5%) | 5,563 (24.5%) | <0.001 |
| **Stomatological** | 20,760 (18.3%) | 11,198 (9.9%) | <0.001 | 5,816 (25.6%) | 3,155 (13.9%) | <0.001 |
| **Cardiovascular system** | 38,744 (34.1%) | 39,729 (35%) | <0.001 | 9,000 (39.6%) | 8,909 (39.2%) | <0.001 |
| **Respiratory system** | 5,313 (4.7%) | 6,301 (5.6%) | <0.001 | 2,352 (10.4%) | 2,289 (10.1%) | <0.001 |
| **All comorbidities** | 54 (0%) | 47 (0%) | 0.86 | 30 (0.2%) | 39 (0.2%) | 0.06 |
