# Supplementary material for: Oral cancer in Hungary: An epidemiological profile (2015–2019)
Source: PLoS One. 2025 Jul 3;20(7):e0327566. doi: 10.1371/journal.pone.0327566 (PMC12225832; doi:10.1371/journal.pone.0327566)
Supplement: S10 Table — (DOCX) [file pone.0327566.s010.docx]

**S10 Table. Comorbidity burden in the case and control group of Hungary from 2015 to 2019 in different genders (percentages as percentages in the relevant population).**

|  | **Control population** | | **p-value** | **Cases population** | | **p-value** |
| --- | --- | --- | --- | --- | --- | --- |
| **Number of comorbidities** | **Male** | **Female** | <0.001 | **Male** | **Female** | <0.001 |
| **0** | 15,160 (13.4%) | 8,766 (7.7%) |  | 1,220 (5.4%) | 620 (2.7%) |  |
| **1** | 21,443 (18.9%) | 14,915 (13.1%) |  | 3,471 (15.3%) | 2,299 (10.1%) |  |
| **2** | 19,550 (17.2%) | 15,200 (13.4%) |  | 4,702 (20.7%) | 3,510 (15.5%) |  |
| **3** | 9,022 (7.9%) | 6,899 (6.1%) |  | 3,064 (13.5%) | 2,357 (10.4%) |  |
| **4** | 1,447 (1.3%) | 1,007 (0.9%) |  | 836 (3.7%) | 553 (2.4%) |  |
| **5** | 63 (0.1%) | 38 (0%) |  | 44 (0.2%) | 26 (0.1%) |  |
| **Total** | 6,6685 (58.7%) | 4,6825 (41.3%) |  | 13,337 (58.7%) | 9,365 (41.3%) |  |
