# Supplementary material for: Oral cancer in Hungary: An epidemiological profile (2015–2019)
Source: PLoS One. 2025 Jul 3;20(7):e0327566. doi: 10.1371/journal.pone.0327566 (PMC12225832; doi:10.1371/journal.pone.0327566)
Supplement: S11 Table — (DOCX) [file pone.0327566.s011.docx]

**S11 Table. Comorbidity burden in the case and control group of Hungary from 2015 to 2019 in different age groups less than 65 years (<65 years) and equal or large than 65 years (65+ years) (percentages as percentages in the relevant population).**

|  | **Control population** | | **p-value** | **Cases population** | | **p-value** |
| --- | --- | --- | --- | --- | --- | --- |
| **Number of comorbidities** | **<65 years** | **65+ years** | <0.001 | <0.001 | **65+ years** | <0.001 |
| **0** | 14,031 (12.4%) | 9,895 (8.7%) |  | 1,382 (6.1%) | 458 (2.0%) |  |
| **1** | 21,302 (18.8%) | 15,056 (13.3%) |  | 3,456 (15.2%) | 2,314 (10.2%) |  |
| **2** | 17,845 (15.7%) | 16,905 (14.9%) |  | 4,340 (19.1%) | 3,872 (17.1%) |  |
| **3** | 7,836 (6.9%) | 8,085 (7.1%) |  | 2,857 (12.6%) | 2,564 (11.3%) |  |
| **4** | 1,209 (1.1%) | 1,245 (1.1%) |  | 781 (3.4%) | 608 (2.7%) |  |
| **5** | 54 (0%) | 47 (0.0%) |  | 48 (0.2%) | 22 (0.1%) |  |
| **Total** | 62,277 (54.9%) | 51,233 (45.1%) |  | 12,864 (56.7%) | 9,838 (43.3%) |  |
